# Electrically regulating nonlinear optical limiting of metal-organic framework film

**DOI:** 10.1038/s41467-022-34139-2

**Published:** 2022-10-26

**Authors:** Zhi-Zhou Ma, Qiao-Hong Li, Zirui Wang, Zhi-Gang Gu, Jian Zhang

**Affiliations:** 1 State Key Laboratory of Structural Chemistry, Fujian Institute of Research on the Structure of Matter, Chinese Academy of Sciences, Fuzhou, Fujian 350002 P. R. China; 2 School of Physical Science and Technology, ShanghaiTech University, Shanghai, 201210 P. R. China; 3 Fujian Science & Technology Innovation Laboratory for Optoelectronic Information of China, Fuzhou, Fujian 350108 P. R. China

**Keywords:** Nonlinear optics, Solid-state chemistry

## Abstract

Regulating nonlinear optical (NLO) property of metal−organic frameworks (MOFs) is of pronounced significance for their scientific research and practical application, but the regulation through external stimuli is still a challenging task. Here we prepare and electrically control the nonlinear optical regulation of conductive MOFs Cu-HHTP films with [001]- (Cu-HHTP_[001]_) and [100]-orientations (Cu-HHTP_[100]_). Z-scan results show that the nonlinear absorption coefficient (*β*) of Cu-HHTP_[001]_ film (7.60 × 10^−6^ m/W) is much higher than that of Cu-HHTP_[100]_ film (0.84 × 10^−6^ m/W) at 0 V and the *β* of Cu-HHTP_[001]_ and Cu-HHTP_[100]_ films gradually increase to 3.84 × 10^−5^ and 1.71 × 10^−6^ m/W at 10 V by increasing the applied voltage, respectively. Due to 2D Cu-HHTP having anisotropy of charge transfer in different orientations, the NLO of MOFs film can be dependent on their growth orientations and improved by tuning the electrical field. This study provides more avenues for the regulation and NLO applications of MOFs.

## Abstract (web summary)

Nonlinear optical properties of metal-organic framework can be tuned for potential optical applications. Here the authors demonstrate an enhancement the nonlinear absorption coefficient of MOF film by applying external electric field.

## Introduction

Metal-organic frameworks (MOFs) are a kind of crystalline network materials made from metal nodes and organic linkers through robust coordination binding^1–3^. Due to their high porosity, large surface area, abundant active sites, designability structures, and tunable functionalities^4–9^, MOFs have attracted great attention in adsorption/separation^10–13^, catalysis^14,15^, biomedicine^16,17^, optics^18–20^, electronics^21,22^ and so on. Especially recently MOFs with π-electronic conjugated systems possess third-order nonlinear optics (NLO), showing potential applications in optical limiting (OL), optical switching, and mode-locked laser systems, etc^23–25^. So far, third-order NLO performances of MOFs have been investigated by adjusting different MOFs structural parameters, including the types of metal chelated ligands^26^, construction of interpenetrated networks^27^, encapsulation of guest species and thickness of MOFs film^23^. However, to expand the practical third-order NLO applications of MOFs, more flexible and direct regulation strategies need to be developed. This calls for the studies to tune the third-order NLO response of MOFs by stimulating external factors, which will help to better understand their NLO performance and expand their optical applications. Particularly, electrical regulation provides a fast and high-efficient strategy for developing new optoelectronic devices and sensors^28–30^. Despite electrical regulation has been used for the study and applications in the fields of materials synthesis^31,32^, adsorption/separation^33,34^, catalysis^35,36^, optical applications^37–41^, electrical regulation of NLO in MOFs has not been reported and is still a challenging task.

Most types of MOFs reported so far have low conductivity, thus limiting their diverse electrical applications^30,42,43^. To achieve efficient electrical tunning of third-order NLO performance, conductive MOFs provide a kind of excellent candidate materials with layered structure and large π-conjugation, which usually are beneficial for high charge mobility and possess good NLO performance^44–48^. Furthermore, compared with powder or bulk MOFs, MOFs films with less light scattering and continuous surface are better for understanding their NLO performance and broadening the practical application^23,26^. In particular, MOFs films prepared by liquid phase epitaxial layer-by-layer (LPE LBL) method (surface-coordinated MOFs thin films, SURMOFs) not only have well-defined high transparency and homogeneous surface, but also have the features of controllable growth orientation and adjustable thickness^49,50^, which exhibit obvious advantage for preparing high-quality films in the NLO regulation and application of MOFs.

Based on the above considerations, in this work, we prepare conductive MOFs Cu-HHTP (HHTP = 2,3,6,7,10,11-hexahydroxytriphenylene) films with [001]- and [100]-orientations by using the LPE LBL method, namely Cu-HHTP_[001]_ and Cu-HHTP_[100]_ films, to study electrically regulating third-order NLO. Z-scan measurement results show that the conductive MOFs with large conjugated systems have strong nonlinear OL. Interestingly, the [001]-orientation has a stronger third-order nonlinear absorption coefficient (*β*) than that of [100]-orientation and the *β* of different orientations increases with different rates via increasing applied voltage due to the anisotropy of charge transfer in different orientations. The *β* of Cu-HHTP_[001]_ film increases from 7.60 × 10^−6^ m/W (0 V) to 3.84 × 10^−5^ m/W (10 V) while that of Cu-HHTP_[100]_ film increases from 0.83 × 10^−6^ m/W (0 V) to 1.71 × 10^−6^ m/W (10 V) with increasing the applied voltage, implying Cu-HHTP_[001]_ has higher increase rate of electrical tunning third-order NLO performance than Cu-HHTP_[100]._ The density functional theory (DFT) calculations also demonstrate the third-order nonlinear response of Cu-HHTP_[001]_ is better than that of Cu-HHTP_[100]_ and can be enhanced by applying the voltages. To the best of our knowledge, this work is the first achievement in the regulation of the nonlinear optical limiting effect by changing the growth orientations and applying the electric voltage in MOFs materials.

## Results

### Preparation of the Cu-HHTP_[001]_ and Cu-HHTP_[100]_ films

**Fig. 1:**
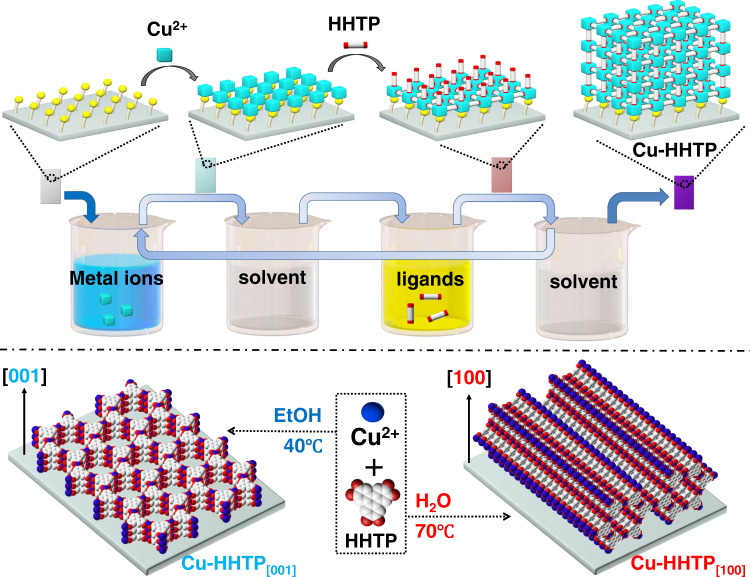
Preparation of Cu-HHTP films. The preparation process of Cu-HHTP films with [001]- and [100]-orientations by LPE LBL method.

In this work, Cu-HHTP films were assembled on the functionalized substrates with LPE LBL autoarm immersion method by sequentially immersing Cu(OAc)_2_ and HHTP solutions (Fig. 1 top). Between each step, the samples were washed to remove the unreacted reactants. Note that two oriented Cu-HHTP films along [001]- and [100]-orientations were prepared by changing the types of solvents and reaction temperature during the preparation process (Fig. 1 bottom). Cu-HHTP_[001]_ film was obtained using ethanol as solvent under 40 °C while Cu-HHTP_[100]_ film was prepared when the solution was deionized water and the reaction temperature was 70 °C. For further studies, ITO glasses were used as the substrates for Cu-HHTP films growth.

### Characterization of the Cu-HHTP_[001]_ and Cu-HHTP_[100]_ films

**Fig. 2:**
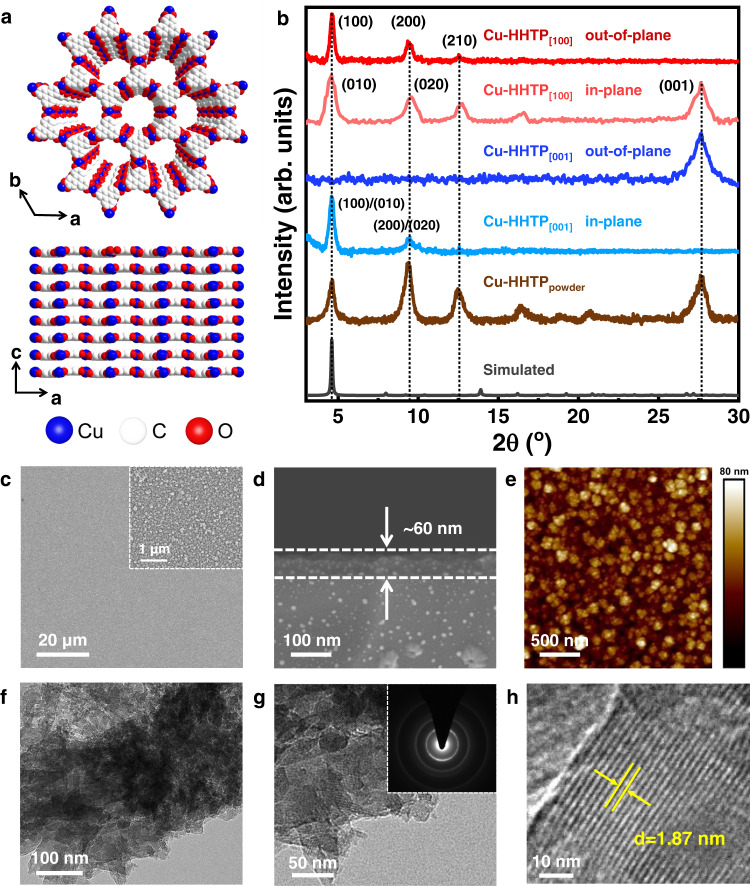
Structural analysis and morphological characterization of Cu-HHTP films. **a** Structural models of Cu-HHTP along c-axis (top) and b-axis (down), H atoms are omitted for clarity. **b** The out-of- and in-plane XRD patterns of Cu-HHTP_[001]_ and Cu-HHTP_[100]_ films, and XRD pattern of Cu-HHTP powder in comparison to the simulated XRD pattern. **c** The surface, **d** cross-sectional SEM and **e** AFM images of Cu-HHTP_[001]_ films. **f** The TEM and **g**, **h** HRTEM images of Cu-HHTP nanosheets scraped from the Cu-HHTP_[001]_ film sample (inserted **g**: SAED pattern). arb. units: arbitrary units.

In the MOFs structure, Cu-HHTP has a graphene-like honeycomb porous and layered structure (Fig. 2a). The crystallinities and growth orientations of Cu-HHTP films were characterized by out-of- and in-plane XRD patterns (Fig. 2b). The out-of-plane XRD pattern of Cu-HHTP_[001]_ film showed a peak at 27.7°, which was identified as (001) peaks of Cu-HHTP. The diffraction peaks at 4.6° and 9.5° in the in-plane XRD pattern were attributed to the (100) and (200) peaks. The diffraction peaks at 4.6°, 9.5°, and 12.6° in the out-of-plane XRD pattern of Cu-HHTP_[100]_ film corresponded to the (100), (200), and (210) peaks of simulated Cu-HHTP, respectively. Its in-plane XRD pattern showed diffraction peaks at 4.6°, 9.5°, and 27.7° corresponding to (010), (020), and (001) peaks. The XRD data showed that Cu-HHTP films with [001]- and [100]-orientations were successfully prepared on substrates.

The morphology and thickness of Cu-HHTP films were characterized by scanning electronic microscopy (SEM). Surface SEM images showed that both Cu-HHTP_[001]_ and Cu-HHTP_[100]_ films had good homogeneity and flat surfaces (Fig. 2c and Supplementary Fig. 1a, b). The cross-sectional SEM images showed that the thickness of Cu-HHTP_[001]_ and Cu-HHTP_[100]_ films were about 60 and 120 nm, respectively (Fig. 2d and Supplementary Fig. 1c). The atomic force microscope (AFM) images of Cu-HHTP_[001]_ and Cu-HHTP_[100]_ films showed that both films had good smooth and continuous surface (Fig. 2e and Supplementary Fig. 1d). In addition, the crystalline phase of Cu-HHTP nanosheets stripped from the sample substrates (Fig. 2f–h and Supplementary Fig. 1e, f) and powder samples prepared by heat capacity method (Supplementary Fig. 2) could be seen in the transmission electron microscope (TEM) images. The TEM images and selected area electron diffractions (SAED) measurements further corroborated the high crystalline phase and revealed an obvious lattice spacing of ~1.87 nm, which was consistent with [100] crystal plane of Cu-HHTP.

IR spectra of Cu-HHTP powder and films showed that the C–O stretching vibration peak shifted from 1217 to 1206 cm^−1^, and the C=C and C=O peaks intensities weakened at 1600 and 1540 cm^−1^ compared with that of ligand HHTP, indicating the coordination between ligand and Cu^2+^ (Supplementary Fig. 3). The Raman spectra of HHTP, Cu-HHTP_powder_, Cu-HHTP_[100]_, and Cu-HHTP_[001]_ films (Supplementary Fig. 4) showed that the OH absorption peaks of Cu-HHTP powder and films at 2881, 3054, and 3197 cm^−1^ and the C=O absorption peaks at 1620 cm^−1^ almost disappeared, which proved that Cu^2+^ was highly coordinated with ligand HHTP. The Cu2*p*, C1*s*, and O1*s* XPS spectra of Cu-HHTP_[001]_ and Cu-HHTP_[100]_ films (Supplementary Figs. 5, 6) showed that Cu, C, and O elements were uniformly distributed in MOFs films.

**Fig. 3:**
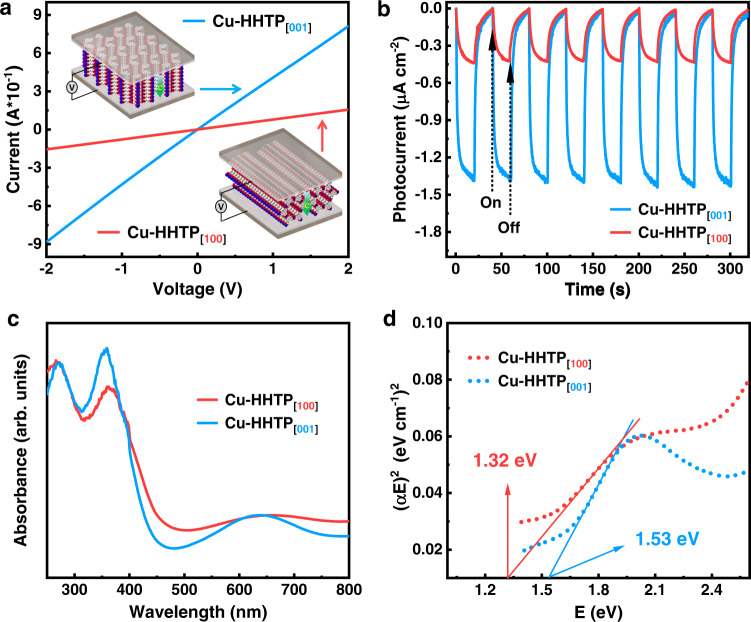
Photoelectrochemical characterization of Cu-HHTP films. **a** *I–V* characteristics with the vertical devices. **b** Photocurrent properties. **c** UV-vis spectra and **d** Tauc plot of Cu-HHTP_[001]_ and Cu-HHTP_[100]_ films. arb. units: arbitrary units.

Since the charge transfer in MOFs films with different orientations has anisotropy, and the charge transfer rate of the interlayer transport path is different from that of the intralayer transport path^51,52^. In order to investigate the conductivity of Cu-HHTP_[001]_ and Cu-HHTP_[100]_ films, the current–voltage (*I–V*) curves were measured by two-probe method and the data showed the conductivity of Cu-HHTP_[001]_ film was better than that of Cu-HHTP_[100]_ film (Fig. 3a). Notably, electrochemical impedance spectroscopy (EIS) showed that both oriented Cu-HHTP_[001]_ and Cu-HHTP_[100]_ thin films were conductivity and the interfacial resistance of Cu-HHTP_[001]_ (77.95 Ω cm^2^) was lower than that of Cu-HHTP_[100]_ (237.74 Ω cm^2^) (Supplementary Fig. 7a, b). Photocurrent measurements (Fig. 3b) revealed that the photocurrent response of Cu-HHTP_[001]_ film was higher than that of Cu-HHTP_[100]_ film, indicating that orientations influence the charge transfer in conductive MOFs and showing different conductivities. Solid-state UV-vis spectra (Fig. 3c) showed there were three characteristic absorption peaks at 269, 356, and 637 nm for Cu-HHTP_[001]_ film, and three peaks at 270, 363, and 644 nm for Cu-HHTP_[100]_ film. The peak at about 270 nm was attributed to the ligand itself, 360 nm could be attributed to the π-π* transition of the MOFs structure, and 640 nm could be attributed to the charge transfer between metal and ligand^53^. Compared to the solid-state UV-vis spectra of Cu-HHTP_[001]_ film, Cu-HHTP_[100]_ film showed a red-shift, which may be related to the orientation of the films. Both Cu-HHTP_[001]_ and Cu-HHTP_[100]_ films showed broad absorption bands in the near-infrared region, and their band gaps were estimated to be 1.53 and 1.32 eV by the Tauc plots, respectively (Fig. 3d).

### The third-order NLO test of the Cu-HHTP_[001]_ and Cu-HHTP_[100]_ films

**Fig. 4:**
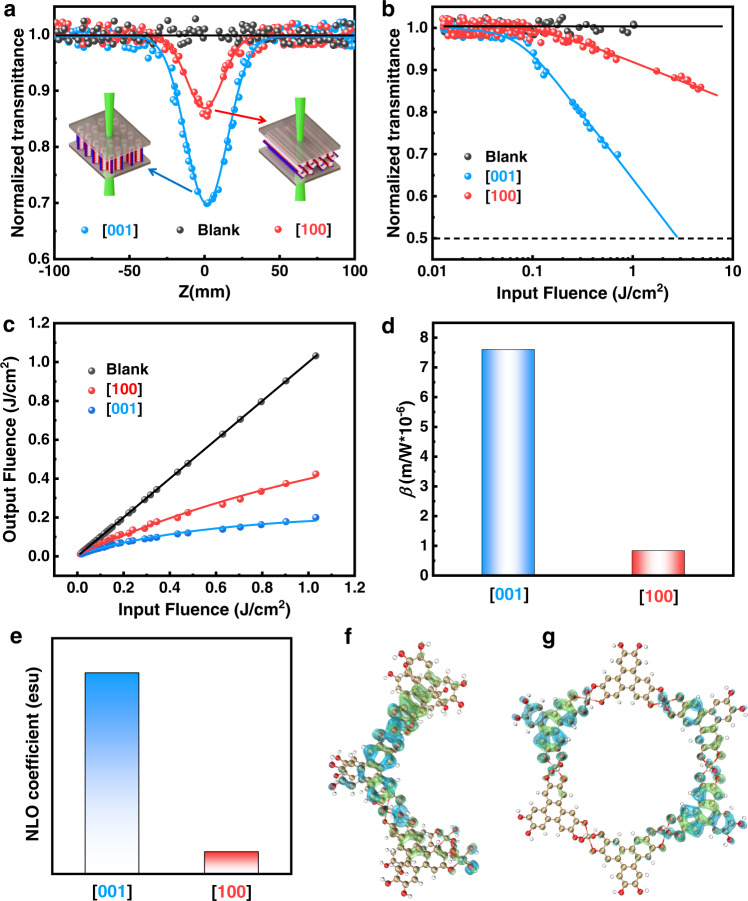
Nonlinear optical limiting performance and theoretical calculation of Cu-HHTP films with different orientations. **a** The open-aperture Z-scan plots of Cu-HHTP_[001]_ and Cu-HHTP_[100]_ films. **b** Variation in the normalized transmittance as a function of input fluence**. c** The curves of output fluence versus input fluence. **d** The third-order nonlinear absorption coefficient (*β*) of Cu-HHTP_[001]_ and Cu-HHTP_[100]_ films. **e** The calculated third-order polarizabilities (*γ*) of Cu-HHTP along [001]- and [100]-orientations. The holes (blue) and electrons (green) distribution models of Cu-HHTP along **f** [001]- and **g** [100]-orientations.

The third-order NLO properties of Cu-HHTP films were tested by using Z-scan technology with a 532 nm nanosecond laser at 80 μJ. The Cu-HHTP_[001]_ and Cu-HHTP_[100]_ films were installed on the computer-controlled translation platform. The thickness of the films could be controlled by optimizing the number of immersion cycles during film preparation, resulting in the linear transmittance of Cu-HHTP_[001]_ and Cu-HHTP_[100]_ films remained at 70% and 61%, respectively (Fig. 4a). The open-aperture Z-scan test for both films showed typical reverse saturation absorption (RSA) responses (Fig. 4a). The minimum normalized transmittances (T_min_) of Cu-HHTP_[001]_ and Cu-HHTP_[100]_ films at Z = 0 were about 0.70 and 0.85, respectively. The bare ITO glass substrate was used as reference. Figure 4b showed the relationship between the normalized transmittance of the films and the laser input fluence. It could be seen that the normalized transmittance of the two films were basically unchanged at low input fluence, and gradually decreased with the increase of input fluence and the decreasing speed of Cu-HHTP_[001]_ film was faster than that of Cu-HHTP_[100]_ film. Figure 4c showed the relationship between the sample laser output fluence and the laser input fluence. Similarly, at low input fluence, the output fluence of the samples showed a high linear relationship. With the further increasing of the laser input fluence, the output fluence began to decay, and then gradually tended to be constant. The decay degree of Cu-HHTP_[001]_ film was higher than that of Cu-HHTP_[100]_ film. As shown in Fig. 4b, c, the NLO curves of Cu-HHTP_[001]_ and Cu-HHTP_[100]_ films showed the typical feature of optical limiting (OL). Figure 4d showed the *β* of the two films after fitting the measured Z-scan curves. The *β* values Cu-HHTP_[001]_ and Cu-HHTP_[100]_ films were calculated to be ~7.60 × 10^−6^ m/W and ~0.84 × 10^−6^ m/W, respectively. These oriented Cu-HHTP films exhibited higher nonlinear OL performance (Supplementary Table 1) than most of reported OL materials and the OL performance could be dependent on the growth orientations, in which [001]-orientation was better than that of [100]-orientation.

To investigate the NLO performance of the MOFs films with different growth orientations, the density of states and third-order polarizabilities (*γ*) of two Cu-HHTP structural models (Fig. 4e and Supplementary Fig. 10a, b and Supplementary Figs. 12, 13) were compared through density functional theory (DFT) calculations. The calculated *γ* for Cu-HHTP along [001]-orientation was 2.36 × 10^−30^ esu, which was much higher than that of Cu-HHTP along [100]-orientation (2.60 × 10^−31^ esu), indicating that the third-order NLO response of Cu-HHTP_[001]_ was stronger than that of Cu-HHTP_[100]_. In addition, the excitation features calculated by time-dependent DFT (Supplementary Fig. 11a, b) showed that the normalized UV-vis spectra of Cu-HHTP along [001] and [100] growth orientations had strong absorption peaks at 672 nm (S_0_-S_98_) and 632 nm (S_0_-S_84_), respectively. The analysis results of electron-hole distributions (Fig. 4f, g) and charge transfer paths of different orientations (Supplementary Table 2) showed that Cu^2+^ and ligands were involved in the composition of holes and electrons in Cu-HHTP along both orientations. However, the electron-hole distribution of Cu-HHTP along [001]-orientation was more concentrated than that of Cu-HHTP along [100]-orientation, which was easy to achieve electron transfer. Consequently, Cu-HHTP along [001]-orientation had multiple electron transfer pathways including metal-ligand charge transfer (9.1%), ligand-ligand charge transfer (36.78%), and local excitation (54.02%) while Cu-HHTP along [100]-orientation was dominated by local excitation (95.13%). Meanwhile, Cu-HHTP along [001]-orientation exists both intralayer (86.21%) and interlayer electron transfer (13.79%) while Cu-HHTP along [100]-orientation only existing intralayer electron transfer (intralayer: 100%), similar to the analysis of density of states. Therefore, the multi-electron transfer pathways of Cu-HHTP along [001]-orientation resulted in a higher third-order NLO response relative to Cu-HHTP along [100]-orientation.

**Fig. 5:**
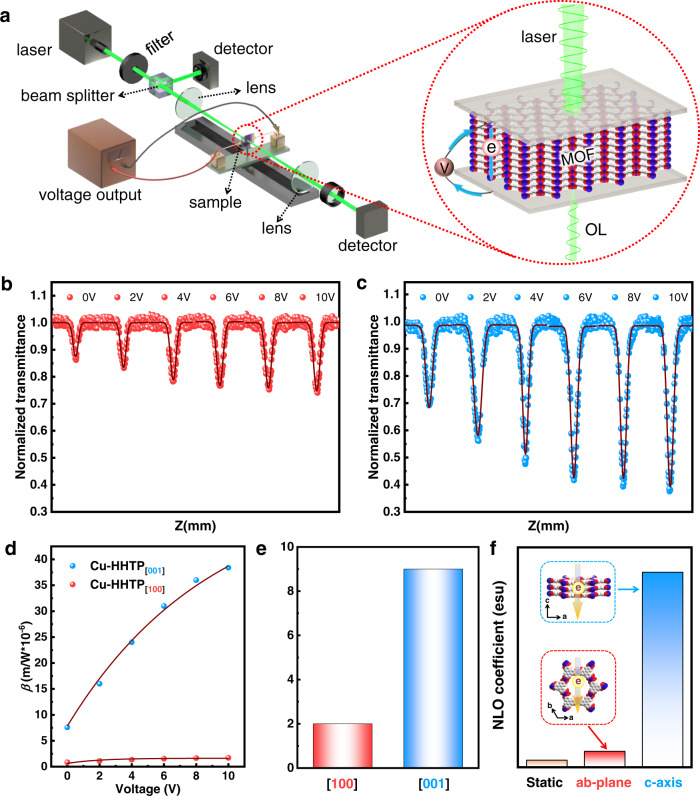
Nonlinear optical limiting performance and theoretical calculation of Cu-HHTP films under external electric field. **a** Schematic diagram of Z-scan setup with external power supply. **b**, **c** The open-aperture plots of the films and **d** the corresponding third-order nonlinear absorption coefficient (*β*) and **e** the R values of Cu-HHTP_[001]_ and Cu-HHTP_[100]_ films with different voltages. **f** The theoretical calculation of third-order polarizabilities (*γ*) comparison for Cu-HHTP along *c*-axis and *ab*-plane by increasing applied voltages and the electron transport model of Cu-HHTP.

As a kind of conductive MOFs film, we tested the NLO performance changes of Cu-HHTP films with different orientations under different external voltage conditions. The experimental setup combining the open-aperture Z-scan system and applied votage apparatus were shown in Fig. 5a. Cu-HHTP films was grown on ITO conductive glass surface by LPE LBL method, and then another ITO glass was attached to MOFs film to obtain “sandwich” ITO-MOFs-ITO for Z-scan test, and the input voltage was adjusted by external power supply. As shown in Fig. 5b, c, the linear transmittances of Cu-HHTP_[001]_ and Cu-HHTP_[100]_ films gradually reduced with the increase of voltage. When the external voltages were 0, 2, 4, 6, 8, and 10 V, the T_min_ of Cu-HHTP_[001]_ film at Z = 0 were 0.70, 0.56, 0.48, 0.41, 0.39, and 0.37, respectively. And the T_min_ of Cu-HHTP_[100]_ film at Z = 0 were 0.85, 0.83, 0.78, 0.76, 0.75 and 0.74, respectively. The corresponding plots of normalized transmittance and output fluence versus input fluence with different voltages of Cu-HHTP_[001]_ and Cu-HHTP_[100]_ films were shown in Supplementary Figs. 8 and 9 respectively. It could be seen that both films exhibited enhanced OL properties under applied voltage conditions.

To quantitatively evaluate the NLO response of Cu-HHTP_[001]_ and Cu-HHTP_[100]_ films with applied voltage, the *β* was calculated by fitting the open-aperture Z-scan curves from Fig. 5b, c, and the values were shown in Fig. 5d. Due to the high voltage can destroy the MOFs, the 0~10 V were applied in both films for the study. The *β* of Cu-HHTP_[001]_ film were calculated to be 7.60 × 10^−6^, 1.61 × 10^−5^, 2.39 × 10^−5^, 3.12 × 10^−5^, 3.62 × 10^−5^, and 3.84 × 10^−5^ m/W for 0, 2, 4, 6, 8 and 10 V, respectively. And for the Cu-HHTP_[100]_ film, when the voltage were 0, 2, 4, 6, 8, and 10 V, the *β* were 0.84 × 10^−6^, 1.10 × 10^−6^, 1.35 × 10^−6^, 1.54 × 10^−6^, 1.66 × 10^−6^, and 1.71 × 10^−6^ m/W, respectively. It is worth noting that Cu-HHTP_[001]_ at 10 V have highest *β* value exceeding other reported NLO materials (Supplementary Table 1). Moreover, compared with Cu-HHTP_[100]_ film, Cu-HHTP_[001]_ film had a higher increase rate of *β*. We used Eq. (1) to quantify the effect of voltage on the *β* values. The variation of *β* for both films conformed to the equation, where R and *β* were positively correlated (Fig. 5e). By calculation, the R values of Cu-HHTP_[001]_ were 9, which was larger than that of the Cu-HHTP_[100]_ film reflecting the calculated *β* growth rate of Cu-HHTP_[001]_ film was greater than that of Cu-HHTP_[100]_ film. The calculated results were in good agreement with the experimental results, showing that the OL effect could be adjusted by adjusting the applied voltage.

Function fitting of the *β* for the film showed that they all conformed to the following equation:

1 $$\beta={\beta }_{0}+{{{{{{\rm{Ae}}}}}}}^{\frac{-V}{R}}$$

Where *β*_0_ is the nonlinear absorption coefficient constant of thin film without the influence of external conditions, A is a constant, R is the changing rate of the film itself affected by voltage and, V is the external applied voltage.

In order to explore the relationship between the electric-field effect and the third-order NLO response, by comparing the two spin modes^54^, a calculation model (Cu_3_L_2_) was constructed (Supplementary Figs. 10c and 14) to investigate the variation of the third-order NLO response of Cu-HHTP with anisotropy before and after voltage application (Fig. 5f). The third-order NLO response increased after applying voltage in *c-*axis ([001]-orientation) and *ab*-plane (containing [100]-orientation). The calculated results showed that the *γ* value were larger than those of Cu-HHTP without voltage (7.33 × 10^−33^ esu). In addition, the *γ* value was 2.01 × 10^−31^ esu when applying voltage in *c-*axis, which was significantly higher than that applying voltage in ab-plane (1.64 × 10^−32^ esu). The calculated results further demonstrated the increased *γ* of [001]-orientation have much higher than that of [100]-orientation, which is consistent with the experimental *β* increase. Due to the electron transfer rate in *c*-axis was higher than that in *ab*-plane, indicating that the transfer of electrons had an impact on the third-order response of Cu-HHTP. The calculation results also illustrated that the NLO property of the conducive MOFs was not only dependent on growth orientations but also greatly influenced by the stimulation of applied voltage, which was consistent with the experimental results.

## Discussion

In summary, we have successfully fabricated [001]- and [100]-oriented Cu-HHTP films by using a LPE LBL dipping method. The Z-scan results showed that conductive Cu-HHTP films has excellent third-order NLO performance and Cu-HHTP_[001]_ film had higher *β* than Cu-HHTP_[100]_ film, as well as their nonlinear optical limiting (OL) performance could be greatly improved by changing the applied voltage. Interestingly, the Cu-HHTP_[001]_ film at 10 V has higest *β* value, which is better than other reported NLO materials. The nonlinear OL of the conductive MOFs films obviously influenced by growth orientations and applying electrical voltage can be attributed to being that the anisotropy of charge transfer in different orientations of 2D conductive Cu-HHTP, which is also confirmed by theoretical DFT calculation of electron-hole distributions and third-order polarizabilities. This study proposes an effective way to tune the third-order NLO properties of MOFs thin film through by changing the growth orientations and stimulating with applied voltages, expanding a meaningful strategy to develop high-performance NLO materials in practical optoelectronic applications.

## Methods

### Materials and instrumentation

All of the chemicals were used after purchase without further purification. The Powder X-ray diffraction (PXRD) analysis were performed on a MiniFlex2 X-ray diffractometer using Cu-Kα radiation (λ = 0.1542 nm) in the 2θ range of 3–30° with a scanning rate of 0.5° min^−1^. IRRAS data were recorded using a Bruker Vertex 70 FTIR spectrometer with 2 cm^−1^ resolution at an angle of incidence of 80° relative to the surface normal. Scanning electron microscope (SEM) images for the morphology of Cu-HHTP films were measured by JSM6700. JEM-2010F was used to record transmission electron microscope (TEM) images. The AFM images were recorded with a Bruker Dimension ICON. X-ray photoelectron spectroscopy (XPS) spectra for the samples were recorded by using an ESCALAB250Xi. The UV-vis spectra for the samples were measured by Lambda 365. Photocurrent measurement was carried out by using a CHI760e electrochemical workstation (Shanghai Chenhua Instrument China).

### Fabrication of Cu-HHTP Powder

A solid mixture of 2,3,6,7,10,11-hexahydroxytriphenylene (HHTP, 7 mg) and Cu(OAc)_2_·2H_2_O (10 mg) was dissolved in 4 mL of deionized water in a 20 mL glass vial and 0.165 mL of 1-methyl-2-pyrrolidone (NMP) was added drop wise. Then, The vial was capped and sonicated for 30 min. The solid mixture was heated at 85 °C for 36 h, followed by natural cooling to room temperature. The resulting black solid mixture was isolated by centrifugation and washed three times by deionized water and acetone. The solid product was dried under vacuum.

### Preparation of functionalized substrates

The ITO glass substrates were firstly cleaned by sequentially ultrasonicating in deionized water, ethanol, acetone. Then, the functionalized substrates were treated with a mixture of concentrated hydrogen peroxide (30%) and NaOH (2 mmol) aqueous solution at a volume ratio of 1:3 was treated at 80 °C for 30 min, then cleaned with deionized water and dried under nitrogen flux for the next preparation.

### Fabrication of Cu-HHTP films with different orientations

The Cu-HHTP films with different orientations were grown on ITO glass substrates using the liquid-phase epitaxy (LPE) layer-by-layer (LbL) method.

#### (1) Fabrication of Cu-HHTP film with [001] orientation(Cu-HHTP_[001]_)

The Cu-HHTP_[001]_ film were fabricated using the following ethanolic solutions: Cu(OAc)_2_•2H_2_O (1 mM) and HHTP (0.015 mM). The functionalized ITO glass substrates were immersed in the solution of Cu(OAc)_2_•2H_2_O for 10 min and then was immersed in HHTP solution for 15 min at 40 °C. Each step was washed with ethanol to remove residual reactants. A total of 10 growth cycles were used for in situ LPE LBL Cu-HHTP_[001]_ film in this work.

#### (2) Fabrication of Cu-HHTP film with [100] orientation(Cu-HHTP_[100]_)

The Cu-HHTP_[100]_ film was fabricated using the following deionized water solutions: Cu(OAc)_2_•2H_2_O (2 mM) and HHTP (0.02 mM). Meanwhile, 10 μL of NMP were added to 100 mL of HHTP solution. The functionalized ITO glass substrates were immersed in the solution of Cu(OAc)_2_•2H_2_O for 10 min and then was immersed in HHTP solution for 15 min at 70 °C. Each step was washed with deionized water to remove residual reactants. A total of 15 growth cycles were used for in situ LPE layer-by-layer Cu-HHTP_[100]_ films in this work.

### Z-scan measurements

The third-order NLO properties of the sample were evaluated by using the Z-scan technique. The excitation light source was an Nd:YAG laser with a repetition rate of 5 Hz. The laser pulse (period, 5 ns; wavelength, 532 nm) was split into two beams with a mirror. The pulse energies at the front and back of the samples were monitored using energy detectors 1 and 2. All of the measurements were conducted at room temperature. The sample was mounted on a computer-controlled translation stage that shifted each sample along the z-axis.

The relationship of the transmission and input laser intensity for a spatially Gaussian beam can be plotted from the open-aperture Z-scan curve. From the input laser pulse energy *E*_in_ and beam radius *ω(z)*, the light fluence *F*_in_*(z)* at any position can be obtained. *F*_in_*(z)* is defined as

$${F}_{{{{{{\rm{in}}}}}}}\left(z\right)=\frac{4{E}_{{{{{{\rm{in}}}}}}}\sqrt{{{{{{\rm{ln}}}}}}2}}{{\pi }^{\frac{3}{2}}{\omega \left(z\right)}^{2}}$$

Where *ω(z)* is defined as:

$$\omega \left(z\right)={\omega }_{0}{\left[1+{\left(\frac{z}{{z}_{0}}\right)}^{2}\right]}^{\frac{1}{2}}$$

where *ω*_0_ (29 μm) and z_0_ are the light beam radius and the Rayleigh range, respectively, and z_0_ is defined as:

$${z}_{0}=\frac{{k\omega }_{0}^{2}}{2}$$

Where *k* is defined as:

$$k=\frac{2{{{{{\rm{\pi }}}}}}}{{{{{{\rm{\lambda }}}}}}}$$

The equation fits for the nonlinear adsorption coefficient *β* as follows:

$$T\left(Z,S=1\right)=\frac{1}{\sqrt{\pi }{q}_{0}\left(Z,0\right)}{\int }_{-{{\infty }}}^{{{\infty }}}{Ln}\left[1+{q}_{0}\left(Z,0\right){e}^{-{r}^{2}}\right]{dr}$$

$${q}_{0}\left(Z,0\right)={\beta I}_{0}{L}_{{{{{{\rm{eff}}}}}}}$$

$${L}_{{{{{{\rm{eff}}}}}}}=\frac{1-{e}^{-\alpha l}}{\alpha }$$

In these equations, *I*_0_ is the on-axis peak intensity at the focus (Z = 0), *L*_eff_ is the effective thickness of the sample, *α* is the linear absorption coefficient, and *l* is the sample thickness.

### DFT calculations

All the calculations were using Gaussian 16. The second hyperpolarizabilities (γ) in this work were used cam-B3LYP functional and 6–31 + g(d,p) basis sets for C, H, O, and Lanl2DZ for Cu with D3 dispersion correction of Grimme. The TDDFT calculations were used cam-B3LYP functional with 6–31 g(d,p) basis sets for C, H, O, and Lanl2DZ for Cu. Multiwfn 3.8(dev) code and VMD software were used to analyse the hyperpolarizabilities and excitation characteristics. The second hyperpolarizabilities were calculated by the coupled perturbed Kohn−Sham method. After the Taylor expansion of the energy (*E*) to the uniform external electric field (**F**) is as follows:

$$E\left({{{{{\bf{F}}}}}}\right)=E\left(0\right)-{\mu }_{0}{{{{{\bf{F}}}}}}-\frac{1}{2}\alpha {{{{{{\bf{F}}}}}}}^{2}-\frac{1}{6}\beta {{{{{{\bf{F}}}}}}}^{3}-\frac{1}{24}\gamma {{{{{{\bf{F}}}}}}}^{4}-\ldots$$

$${{{{{\rm{\mu }}}}}}\left({{{{{\bf{F}}}}}}\right)=-\frac{\partial E}{\partial {{{{{\boldsymbol{F}}}}}}}={\mu }_{0}+\alpha {{{{{\bf{F}}}}}}+\left(\frac{1}{2}\right)\beta {{{{{{\bf{F}}}}}}}^{2}+\left(\frac{1}{6}\right)\gamma {{{{{{\bf{F}}}}}}}^{3}+\ldots$$

The *i* components of γ are defined as

$${{{{{{\rm{\gamma }}}}}}}_{i}=\left(\frac{1}{15}\right)\mathop{\sum}\limits_{j}({{{{{{\rm{\gamma }}}}}}}_{ijji}+{{{{{{\rm{\gamma }}}}}}}_{ijij}+{{{{{{\rm{\gamma }}}}}}}_{iijj})\,i,j=\{x,y,z\}$$

The total magnitude of γ is measured as

$${{{{{{\rm{\gamma }}}}}}}_{tot}=\sqrt{{{{{{{\rm{\gamma }}}}}}}_{x}^{2}+{{{{{{\rm{\gamma }}}}}}}_{y}^{2}+{{{{{{\rm{\gamma }}}}}}}_{z}^{2}}$$

### Reporting summary

Further information on research design is available in the Nature Research Reporting Summary linked to this article.

## Supplementary information

Supplementary Information

Reporting Summary

## Data Availability

The authors declare that all relevant data are available in this paper and its Supplementary Information and that data supporting the results of this study are available from corresponding authors upon reasonable request.
