# Supplementary material for: Electrically regulating nonlinear optical limiting of metal-organic framework film
Source: Nat Commun. 2022 Oct 26;13:6347. doi: 10.1038/s41467-022-34139-2 (PMC9606303; doi:10.1038/s41467-022-34139-2)
Supplement: Supplementary file 1 — Supplementary Information [file 41467_2022_34139_MOESM1_ESM.pdf]

Supporting Information

Electrically Regulating Nonlinear Optical Limiting of Metal-Organic Framework Film

Zhi-Zhou Ma[a], *Qiao-Hong Li*[a], *Zirui Wang*[ab], *Zhi-Gang Gu*[ac]* *and Jian Zhang*[ac]

[a]State Key Laboratory of Structural Chemistry, Fujian Institute of Research on the Structure of Matter, Chinese Academy of Sciences, Fuzhou, Fujian 350002, P. R. China.

[b]School of Physical Science and Technology, ShanghaiTech University, Shanghai 201210, P.R. China.

[c]Fujian Science & Technology Innovation Laboratory for Optoelectronic Information of China, Fuzhou, Fujian 350108, P. R. China.

*Corresponding author: zggu@fjirsm.ac.cn.

Table of Content

1. Supplementary Figures
**Supplementary Fig. 1.** The surface (a, b), cross-sectional (c) SEM images and AFM images (d) of Cu-HHTP$_{[100]}$ film; The TEM (e, insert: SAED image) and HRTEM images (f) of Cu-HHTP scraped from Cu-HHTP$_{[100]}$ films.
**Supplementary Fig. 2.** TEM (a) and HRTEM (b) as well as SAED images of Cu-HHTP$_{powder}$.
**Supplementary Fig. 3.** IR spectra of HHTP ligand, Cu-HHTP$_{powder}$, Cu-HHTP$_{[001]}$ and Cu-HHTP$_{[100]}$ film. arb. units: arbitrary units.
**Supplementary Fig. 4.** Raman spectra of HHTP ligand, Cu-HHTP$_{powder}$, Cu-HHTP$_{[001]}$ and Cu-HHTP$_{[100]}$ films. arb. units: arbitrary units.
**Supplementary Fig. 5.** XPS spectra of Cu-HHTP$_{[001]}$ film: (a) XPS survey scan spectra; (b) The Cu 2*p* XPS spectra; (c) C1*s* XPS spectra; (d) O1*s* XPS spectra. arb. units: arbitrary units.
**Supplementary Fig. 6.** XPS spectra of Cu-HHTP$_{[100]}$ film: (a) XPS survey scan spectra; (b) The Cu 2*p* XPS spectra; (c) C1*s* XPS spectra; (d) O1s XPS spectra. arb. units: arbitrary units.
**Supplementary Fig. 7.** The EIS spectra for Cu-HHTP$_{[001]}$ (a) and Cu-HHTP$_{[100]}$ (b) film.
**Supplementary Fig. 8.** (a) The corresponding plots of normalized transmittance versus input fluence for Cu-HHTP$_{[001]}$ film with different voltages; (b) The curves of output fluence versus input fluence for Cu-HHTP$_{[001]}$ film with different

voltages.
**Supplementary Fig. 9.** (a) The corresponding plots of normalized transmittance versus input fluence for Cu-$HHTP_{[100]}$ film with different voltages; (b) The curves of output fluence versus input fluence for Cu-$HHTP_{[100]}$ film with different voltages.
**Supplementary Fig. 10.** Simulation structural models of Cu-HHTP along [001]-orientation (a), [100]-orientation (b); and simple model (Cu_3L_2) of applied voltage(c) for theoretical calculation, respectively.
**Supplementary Fig. 11.** Calculated UV-vis absorption spectra of Cu-HHTP along [001]-orientation (a), [100]-orientation (b), respectively.
**Supplementary Fig. 12.** The simulation structural models and molecular orbitals of [001]-orientation (a) and [100]-orientation (b) based on DFT calculations (iso=0.02).
**Supplementary Fig. 13.** DOS plots of [001]-orientation (a) and [100]-orientation (b).
**Supplementary Fig. 14.** The spin analysis of Cu_3L_2**.**
**Supplementary Table 1.** The nonlinear absorption coefficient (β) comparison of some reported NLO materials and the presented Cu-HHTP film.
**Supplementary Table 2.** The main excited state characteristics. (MMCT: metal-metal charge transfer; MLCT: metal-ligand charge transfer; LLCT: ligand-ligand charge transfer; LE: local excitation.)

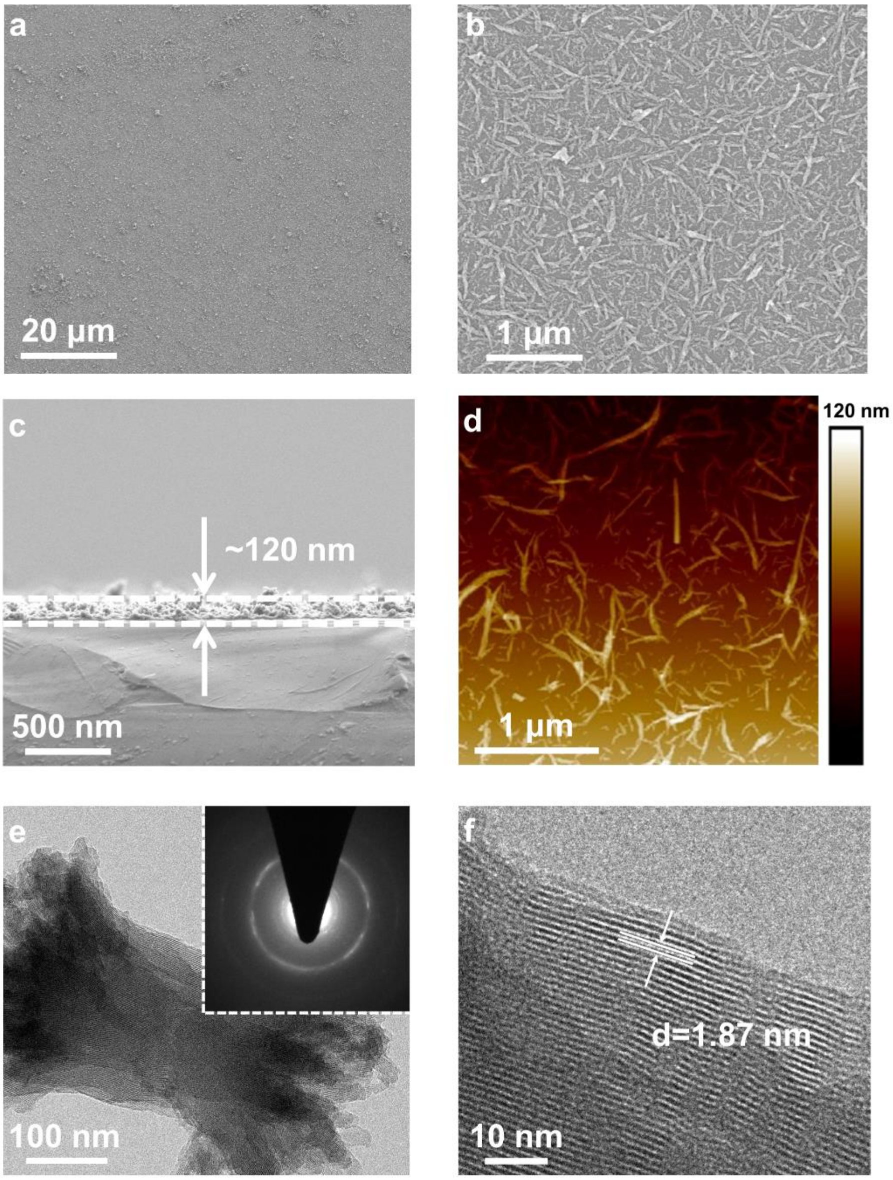

Supplementary Fig. 1. The surface (a, b), cross-sectional (c) SEM images and AFM images (d) of Cu-HHTP$_{[100]}$ film; The TEM (e, insert: SAED image) and HRTEM images (f) of Cu-HHTP scraped from Cu-HHTP$_{[100]}$ films.

Cu-HHTP$_{powder}$.

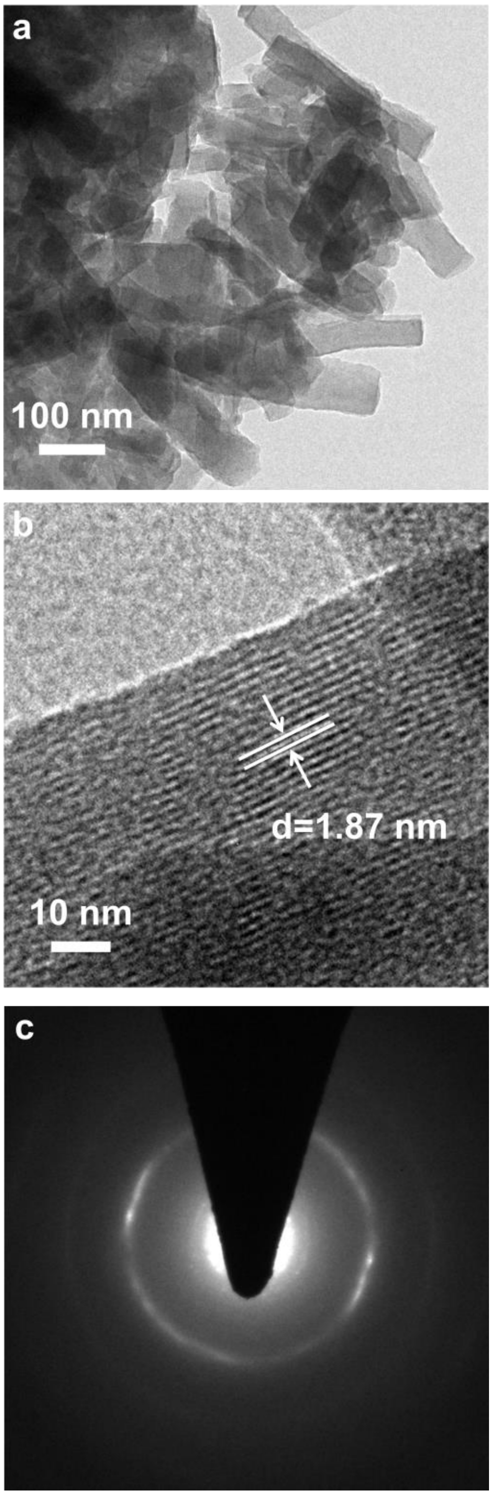

Supplementary Fig. 2. TEM (a) and HRTEM (b) as well as SAED (c) images of Cu-HHTP$_{powder}$.

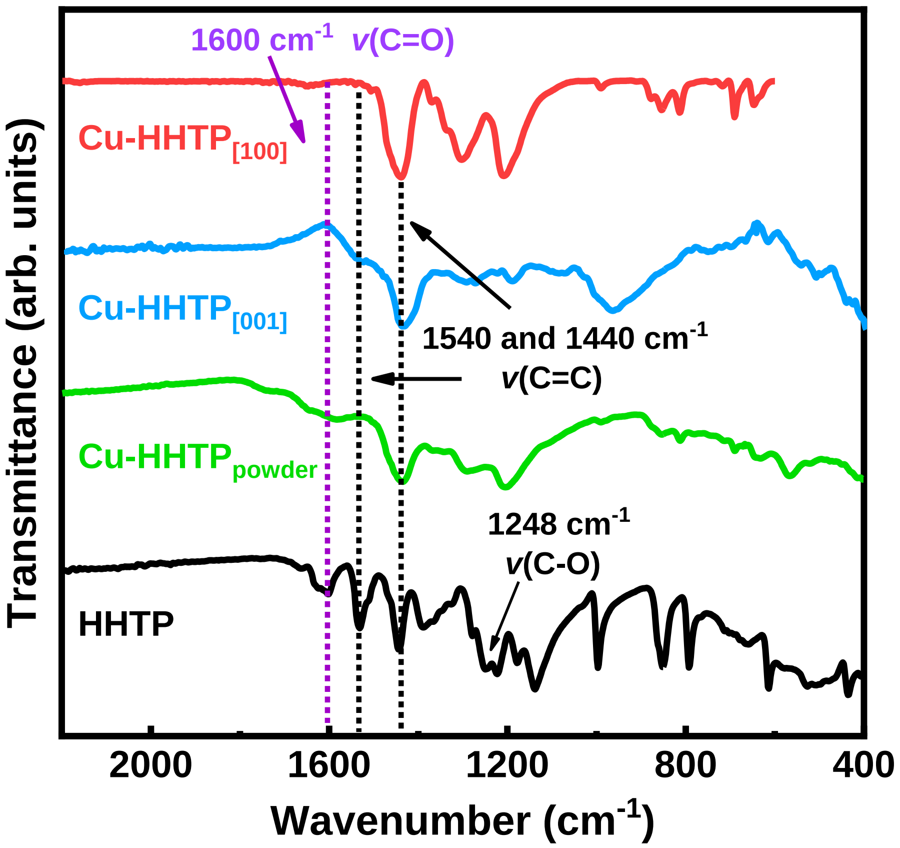

Supplementary Fig. 3. IR spectra of HHTP ligand, Cu-$HHTP_{powder}$, Cu-$HHTP_{[001]}$ and Cu-$HHTP_{[100]}$ film. arb. units: arbitrary units.

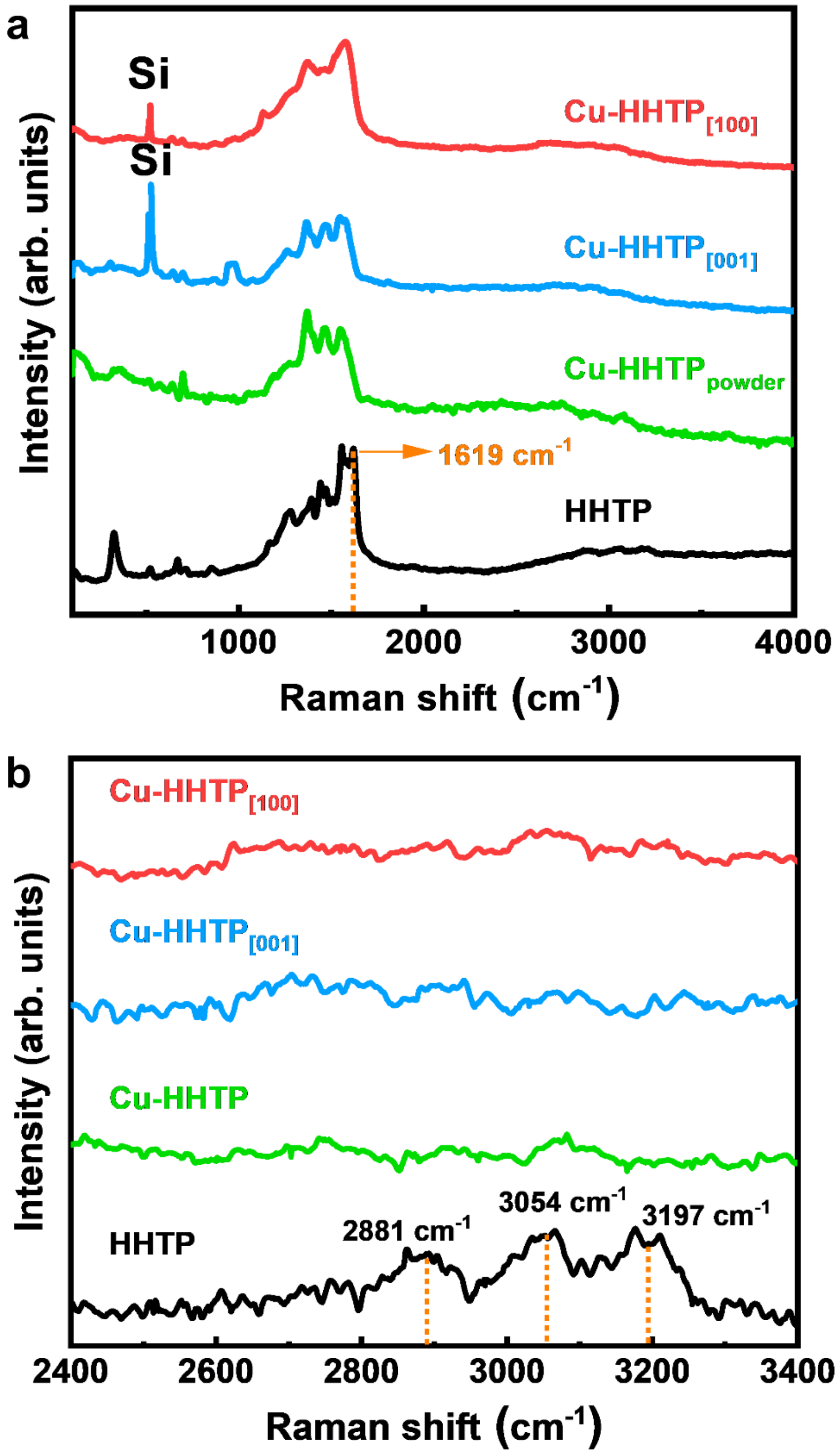

Supplementary Fig. 4. Raman spectra of HHTP ligand, Cu-HHTP$_{powder}$, Cu-HHTP$_{[001]}$ and Cu-HHTP$_{[100]}$ films. arb. units: arbitrary units.

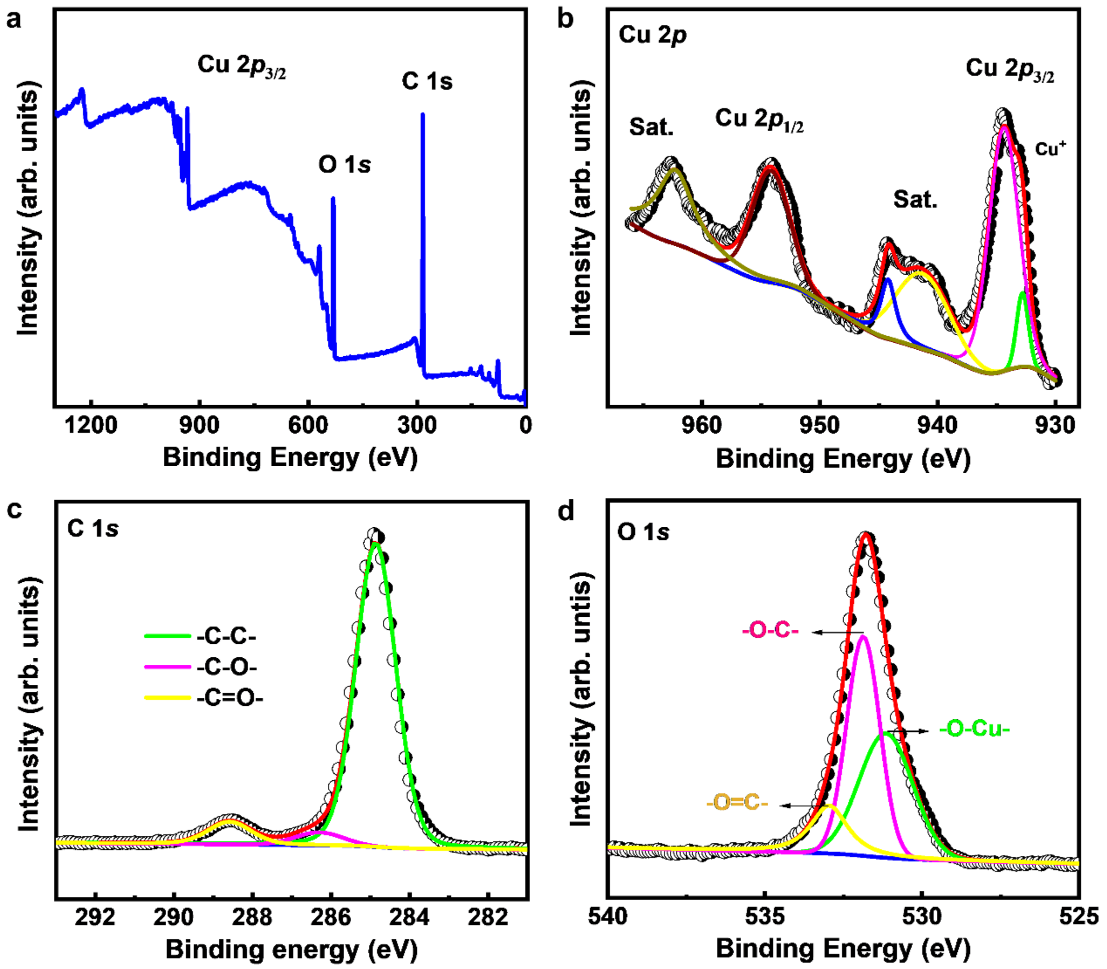

Supplementary Fig. 5. XPS spectra of Cu-HHTP$_{[001]}$ film: (a) XPS survey scan spectra; (b) The Cu 2*p* XPS spectra; (c) C1*s* XPS spectra; (d) O1*s* XPS spectra. arb. units: arbitrary units.

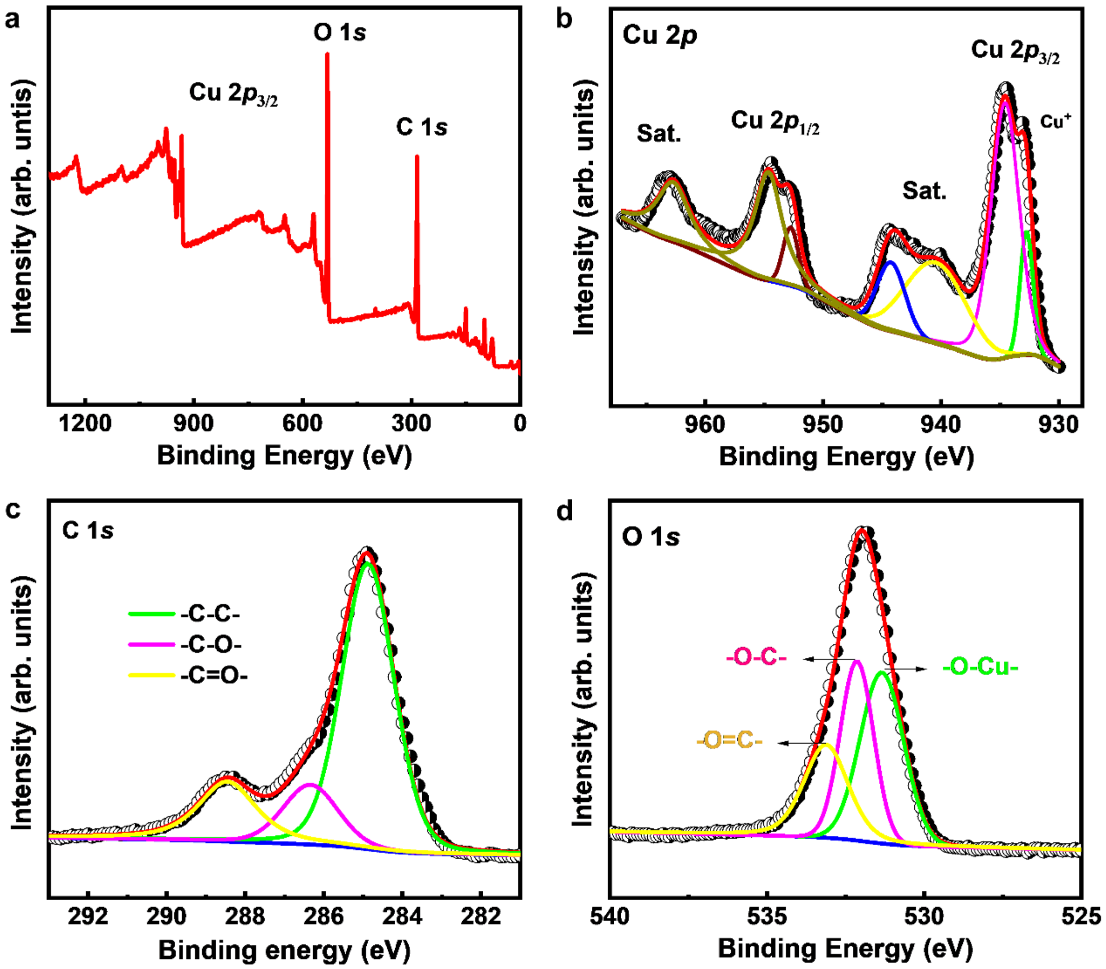

Supplementary Fig. 6. XPS spectra of Cu-HHTP$_{[100]}$ film: (a) XPS survey scan spectra; (b) The Cu 2*p* XPS spectra; (c) C1*s* XPS spectra; (d) O1*s* XPS spectra. arb. units: arbitrary units.

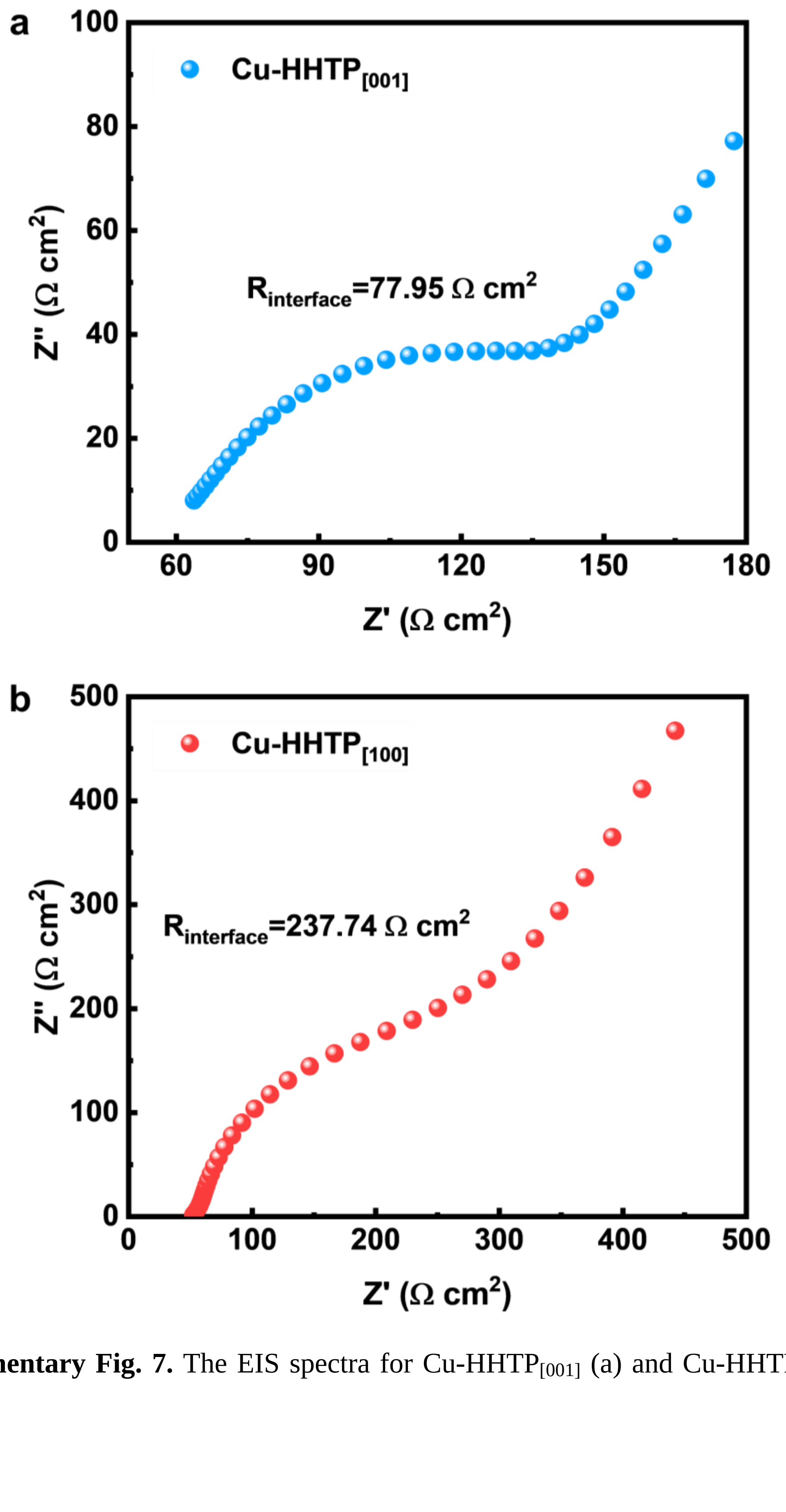

Supplementary Fig. 7. The EIS spectra for Cu-HHTP$_{[001]}$ (a) and Cu-HHTP$_{[100]}$ (b) film.

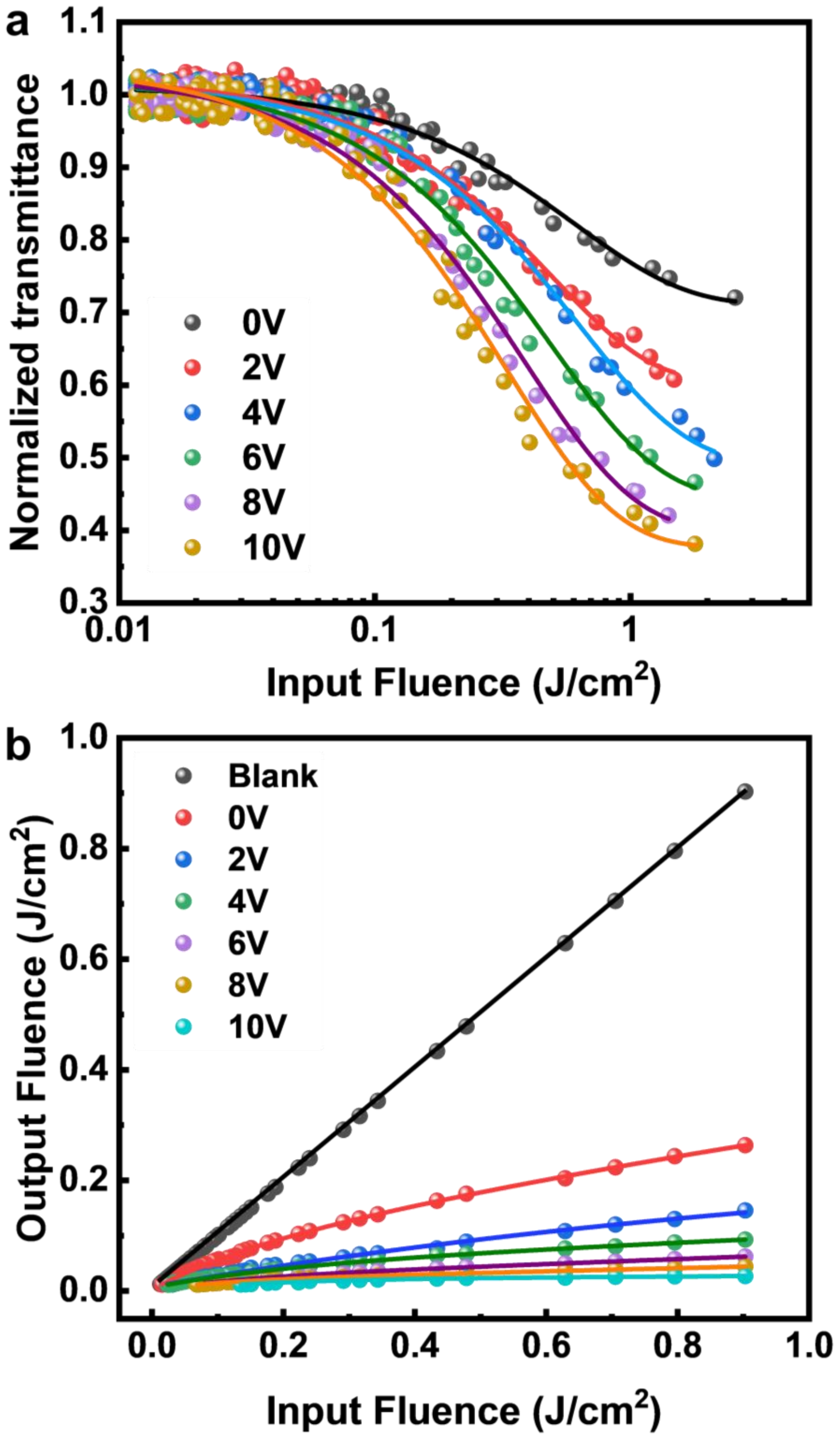

Supplementary Fig. 8. (a) The corresponding plots of normalized transmittance versus input fluence for Cu-HHTP$_{[001]}$ film with different voltages; (b) The curves of output fluence versus input fluence for Cu-HHTP$_{[001]}$ film with different voltages.

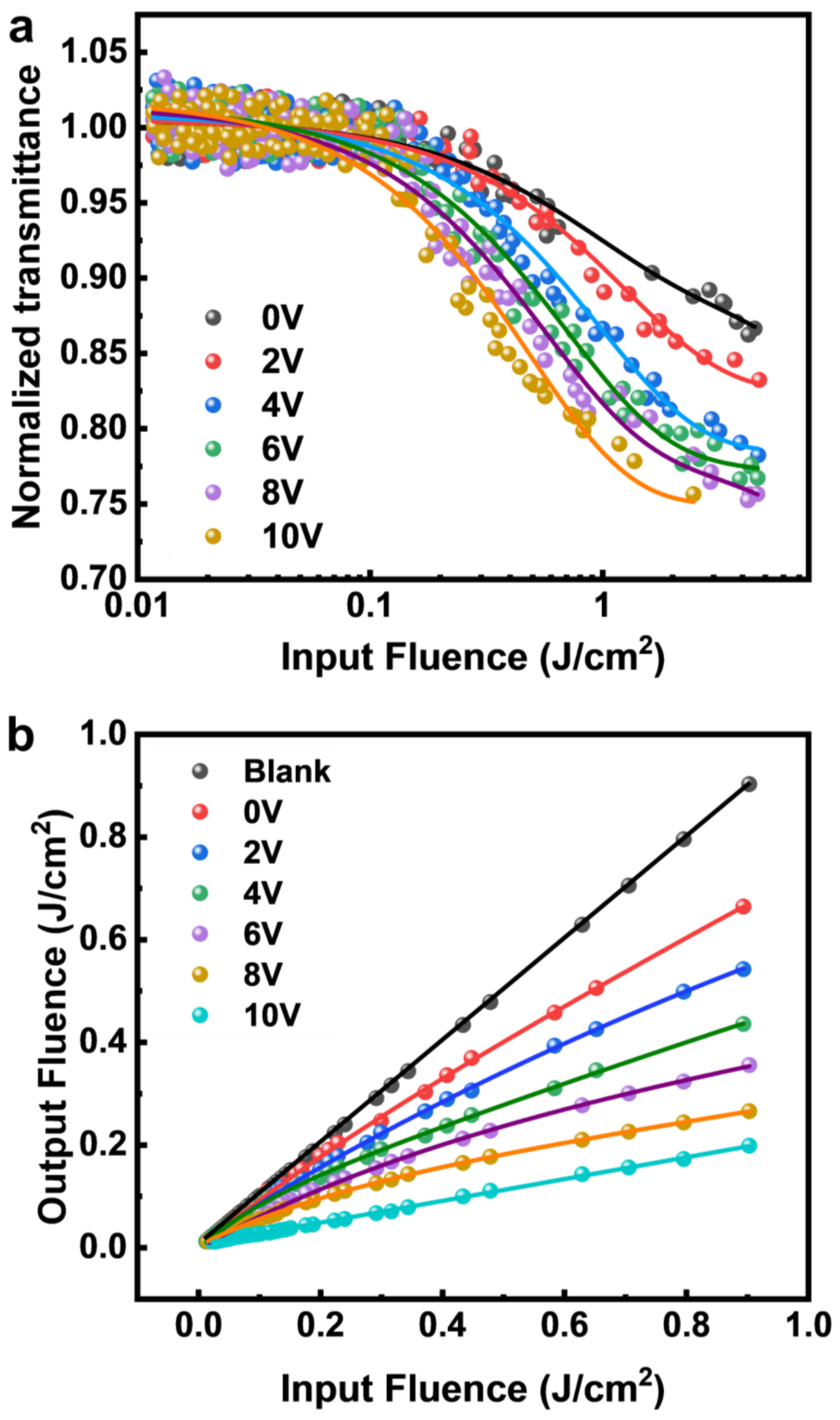

Supplementary Fig. 9. (a) The corresponding plots of normalized transmittance versus input fluence for Cu-HHTP$_{[100]}$ film with different voltages; (b) The curves of output fluence versus input fluence for Cu-HHTP$_{[100]}$ film with different voltages.

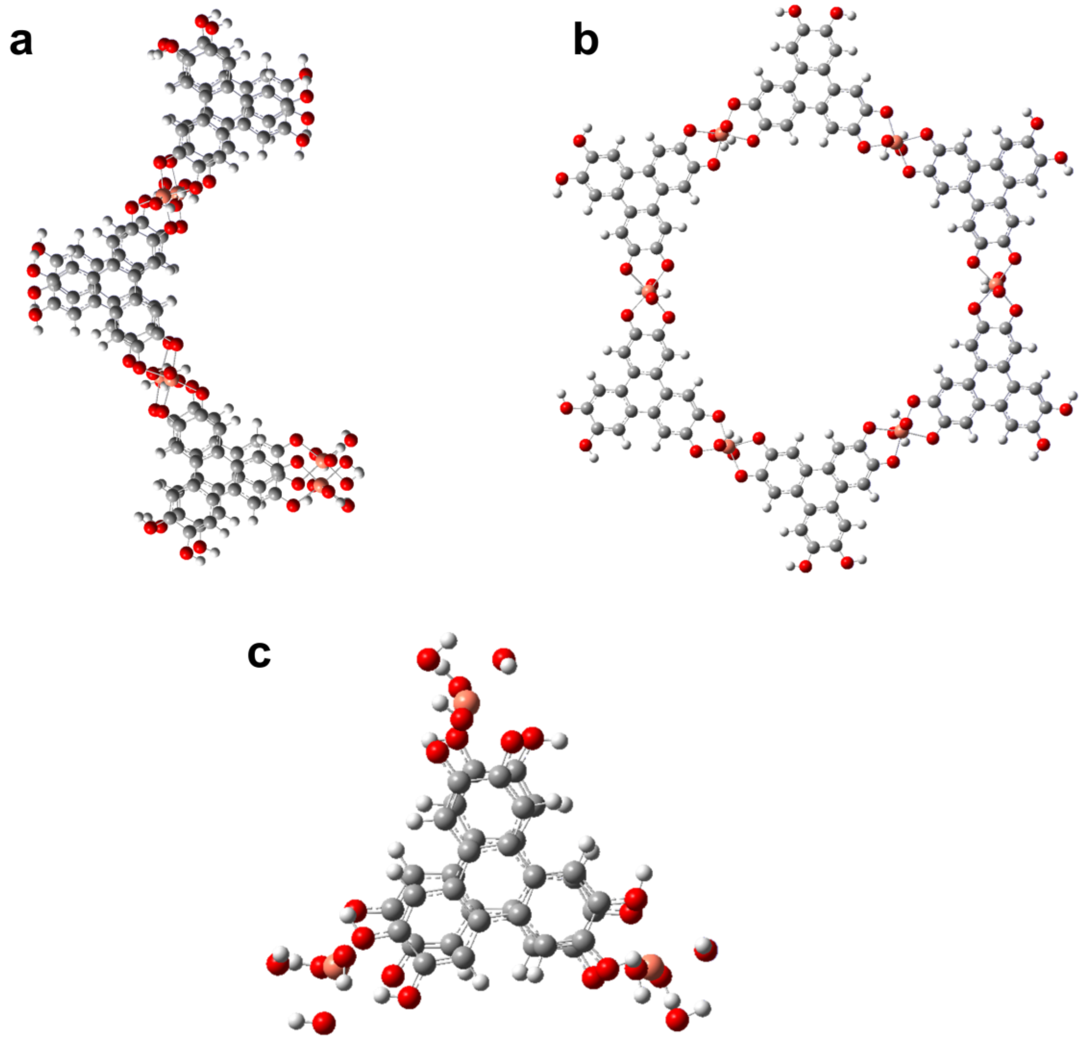

Supplementary Fig. 10. Simulation structural models of Cu-HHTP along [001]-orientation (a), [100]-orientation (b); and simple model (Cu_3L_2) of applied voltage(c) for theoretical calculation, respectively.

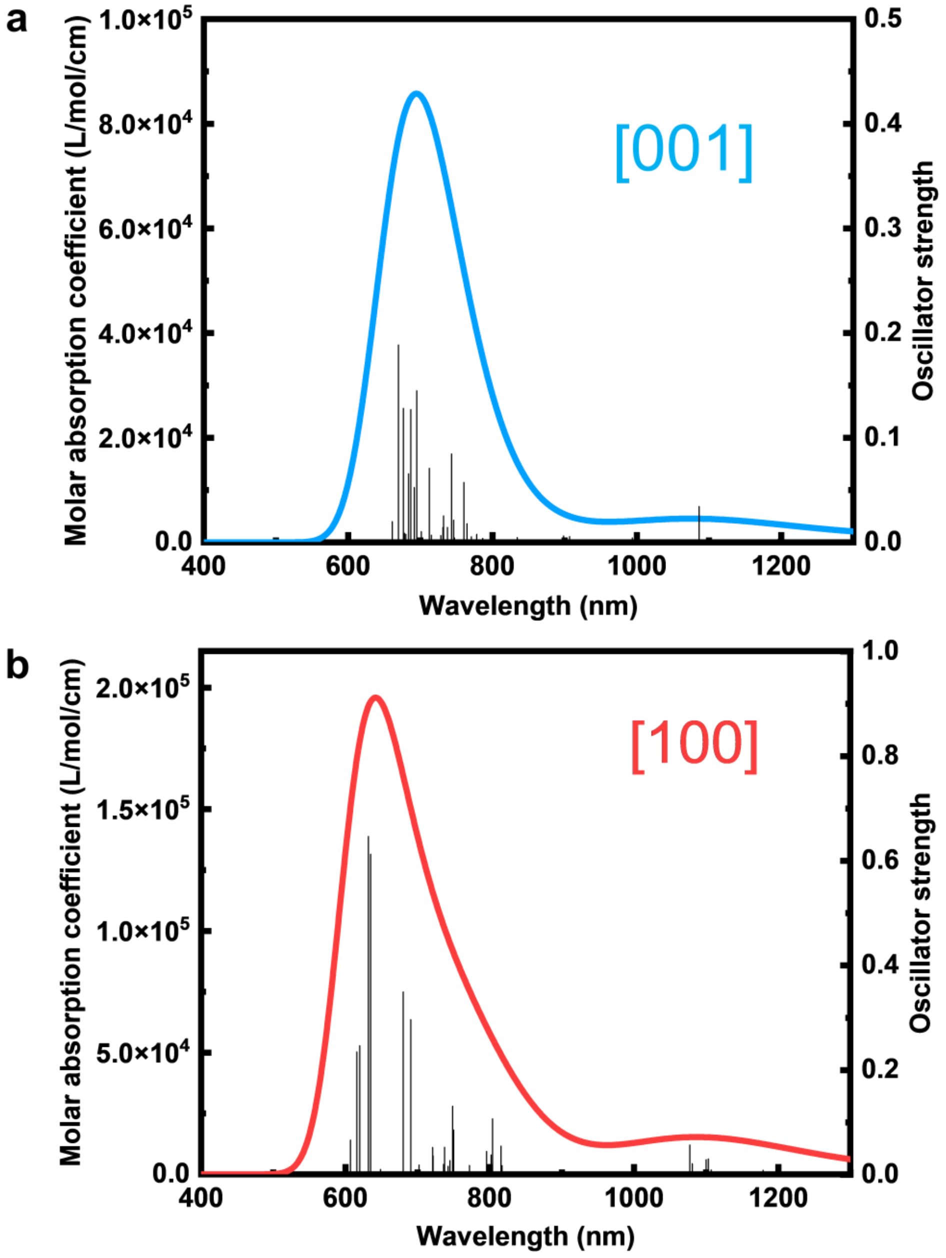

Supplementary Fig. 11. Calculated UV-vis absorption spectra of Cu-HHTP along [001]-orientation (a), [100]-orientation (b), respectively.

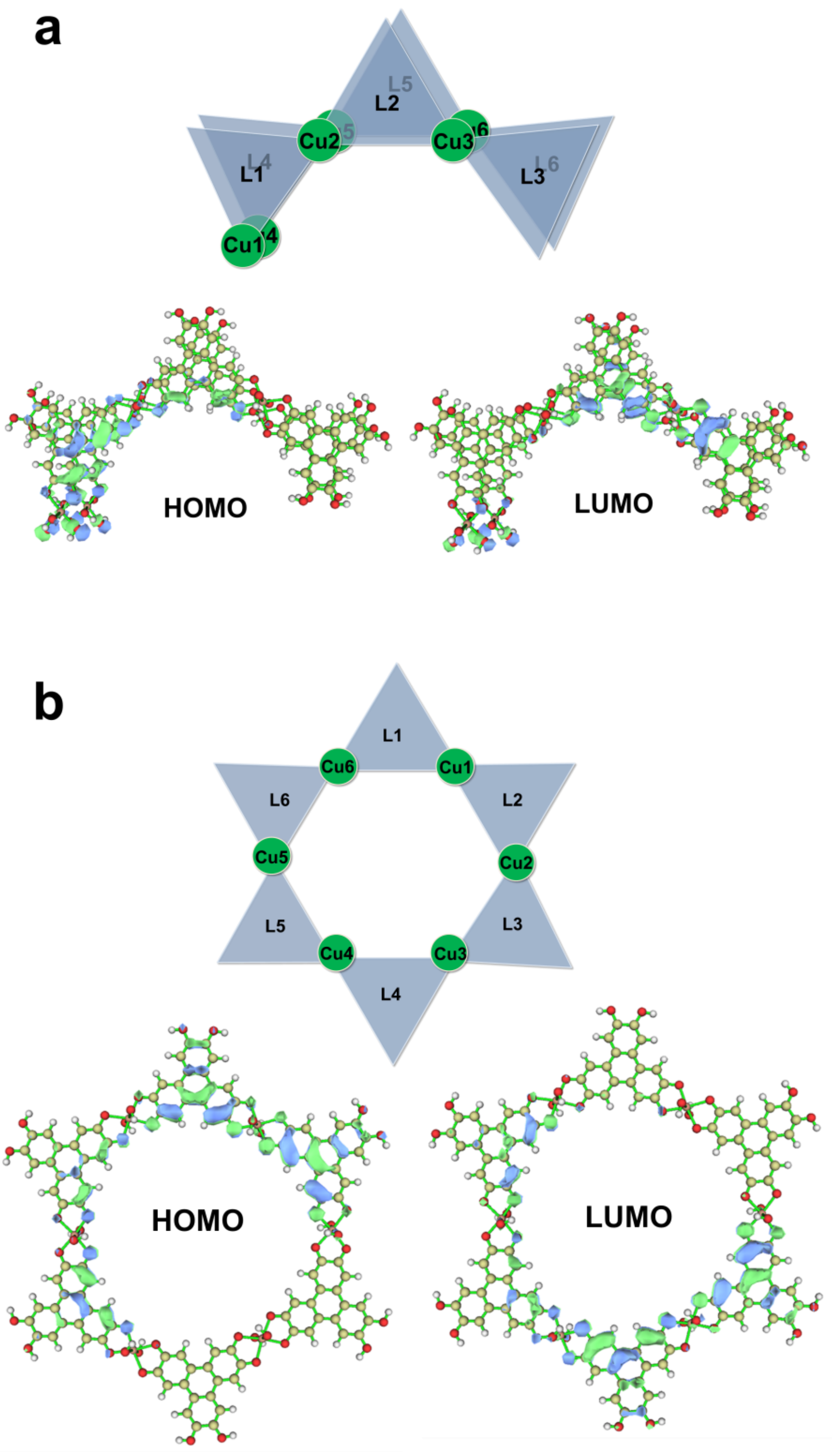

Supplementary Fig. 12. The simulation structural models and molecular orbitals of [001]-orientation (a) and [100]-orientation (b) based on DFT calculations (iso=0.02).

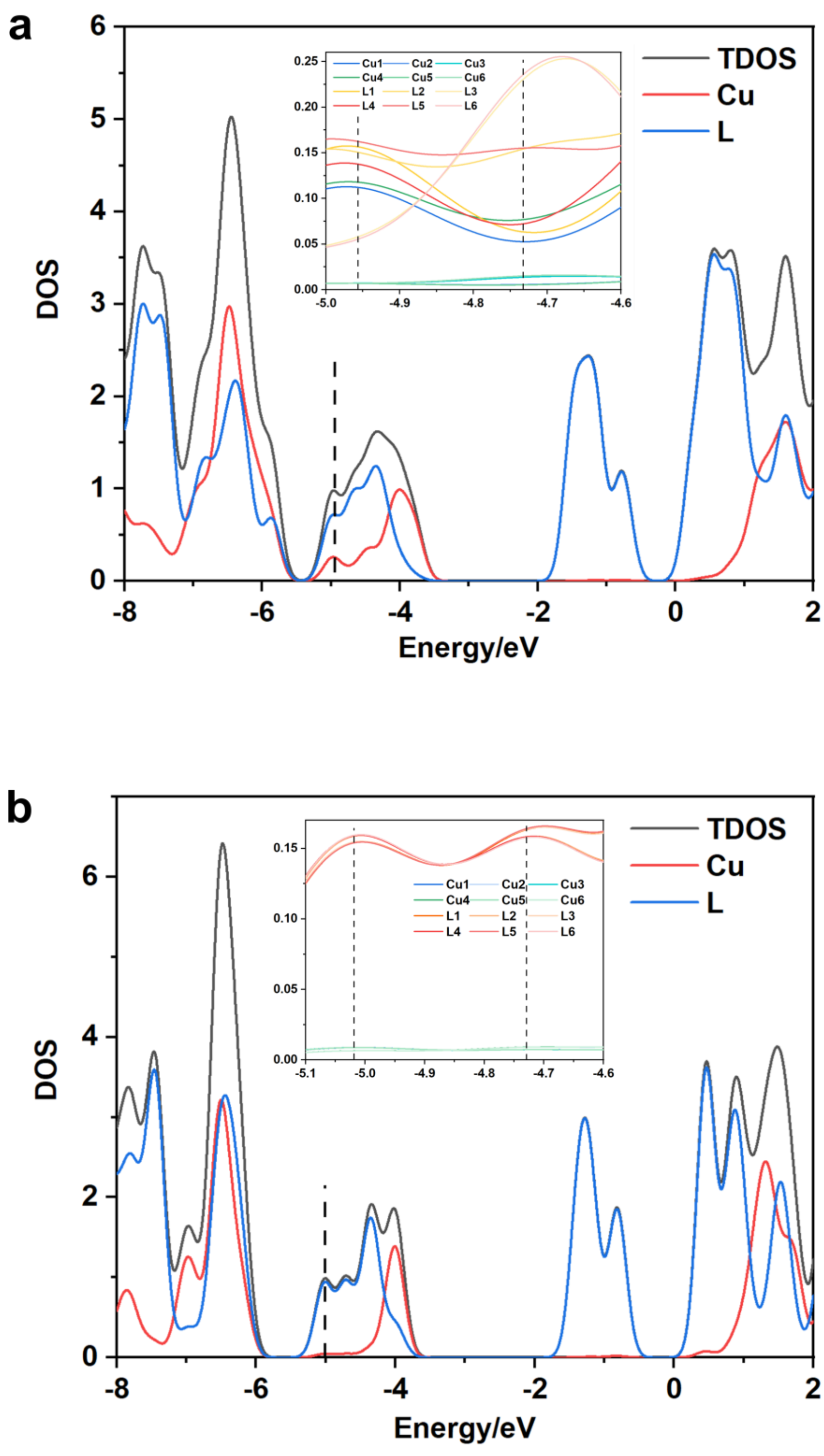

Supplementary Fig. 13. DOS plots of [001]-orientation (a) and [100]-orientation (b).

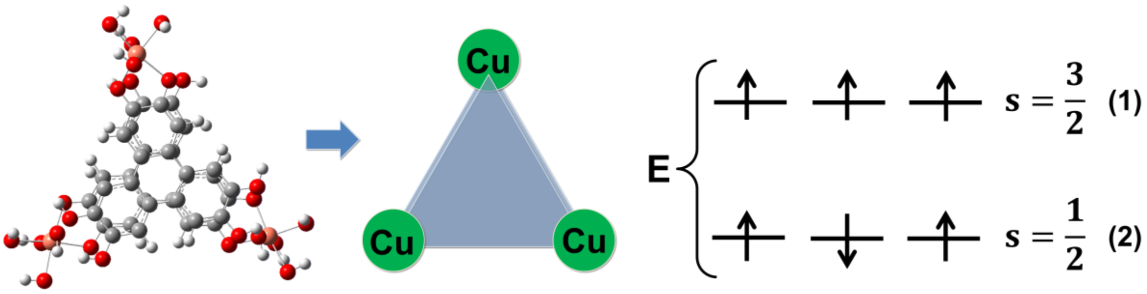

Supplementary Fig. 14. The spin analysis of Cu_3L_2**.**

Supplementary Table 1. The nonlinear absorption coefficient (β) comparison of some reported NLO materials and the presented Cu-HHTP films.

**Samples**	**β (m/W)**	**References**
Cu-HHTP$_{[001]}$ film (0 V)	~7.60×10^{-6}	**This work**
Cu-HHTP$_{[001]}$ film (2 V)	~1.61×10^{-5}	**This work**
Cu-HHTP$_{[001]}$ film (4 V)	~2.39×10^{-5}	**This work**
Cu-HHTP$_{[001]}$ film (6 V)	~3.12×10^{-5}	**This work**
Cu-HHTP$_{[001]}$ film (8 V)	~3.62×10^{-5}	**This work**
Cu-HHTP$_{[001]}$ film (10 V)	**~3.84×10^{-5}**	**This work**
Cu-HHTP$_{[100]}$ film (0 V)	~0.84×10^{-6}	**This work**
Cu-HHTP$_{[100]}$ film (2 V)	~1.10×10^{-6}	**This work**
Cu-HHTP$_{[100]}$ film (4 V)	~1.35×10^{-6}	**This work**
Cu-HHTP$_{[100]}$ film (6 V)	~1.54×10^{-6}	**This work**
Cu-HHTP$_{[100]}$ film (8 V)	~1.66×10^{-6}	**This work**
Cu-HHTP$_{[100]}$ film (10 V)	~1.71×10^{-6}	**This work**
ZnTPyP-1	~3.61×10^{-5}	**1**
ZnTPyP-2	~1.12×10^{-5}	
ZnTPyP-1(Zn/Cu)/PDMS	~4.65×10^{-8}	
ZnTPyP-1(Zn/Mn)/PDMS	~3.10×10^{-8}	
ZnTPyP-1(Zn/Zn)/PDMS	~1.35×10^{-8}	
ZnTPyP-2/PDMS	~4.00×10^{-9}	
ZnTPyP(Cu) film	~5.70×10^{-6}	**2**
ZnTPyP(Ni) film	~1.80×10^{-6}	
ZnTPyP(Mn) film	~1.20×10^{-6}	
ZnTPyP(H_2) film	~5.10×10^{-7}	
PIZA-1 thin film	1.90×10^{-6}	**3**

C_{60}@PIZA-1 thin film	2.80×10^{-6}	
Por-TzTz-POF	1.10×10^{-8}	**4**
Por-COF-HH	1.04×10^{-8}	**5**
Por-COF-ZnNi	4.17×10^{-8}	
Por-COF-ZnCu	4.47×10^{-8}	
MoS_2/PMMA	9.70×10^{-9}	**6**
MoS_2-pvk	9.17×10^{-9}	**7**
Pure grapheme	9.00×10^{-9}	**8**
Zinc porphyrin	3.66×10^{-9}	
Copper porphyrin	1.32×10^{-9}	
PFTP-RGO/PMMA	2.96×10^{-9}	**9**
BP:C_{60}	2.41×10^{-9}	**10**
Cu-HHTP Powder	1.33×10^{-9}	**11**
Co-THPP (ultrathin films)	9.50×10^{-10}	**12**
Zn-THPP	4.60×10^{-10}	**13**
PF-RGO	7.07×10^{-11}	**14**
Pt-Ni cluster/rGO	1.98×10^{-11}	**15**
3D tetrazine chromophore MOFs	$(2.8\text{-}4.6)\times10^{-12}$	**16**

Supplementary Table 2. The main excited state characteristics. (MMCT: metal-metal charge transfer; MLCT: metal-ligand charge transfer; LLCT: ligand-ligand charge transfer; LE: local excitation.)

	**%MMCT**	**%MLCT**	**%LLCT**	**%LE**	**%Intralayer**	**%Interlayer**
001：S_{98}	0.10%	9.10%	36.78%	54.02%	86.21%	13.79%
100：S_{84}	0.00%	0.82%	4.05%	95.13%	100%	0%

References

1. Li, D.-J. et al. Interpenetrated metal-porphyrinic framework for enhanced nonlinear optical limiting. *J. Am. Chem. Soc.* **143**, 17162-17169 (2021).

2. Li, D.-J., Li, Q.-h., Gu, Z.-G. & Zhang, J. Oriented assembly of 2D metal-pyridylporphyrinic framework films for giant nonlinear optical limiting. *Nano. Lett.* **21**, 10012-10018 (2021).

3. Li, D.-J., Gu, Z.-G. & Zhang, J. Auto-controlled fabrication of a metal-porphyrin framework thin film with tunable optical limiting effects. *Chem. Sci.* **11**, 1935-1942 (2020).

4. Samal, M. et al. A thiazolo 5,4-d thiazole-bridged porphyrin organic framework as a promising nonlinear optical material. *Chem. Commun.* **55**, 11025-11028 (2019).

5. Biswal, B. P. et al. Nonlinear optical switching in regioregular porphyrin covalent organic frameworks. *Angew. Chem. Int. Ed.* **58**, 6896-6900 (2019).

6. Liang, G. et al. Optical limiting properties of a few-layer MoS2/PMMA composite under excitation of ultrafast laser pulses. *J. Mater. Chem. C.* **7**, 495-502 (2019).

7. Cheng, H. et al. Covalent modification of MoS_2 with poly(N-vinylcarbazole) for solid-state broadband optical limiters. *Chem. Eur. J.* **22**, 4500-4507 (2016).

8. Krishna, M. B. M., Kumar, V. P., Venkatramaiah, N., Venkatesan, R. & Rao, D. N. Nonlinear optical properties of covalently linked graphene-metal porphyrin composite materials. *Appl. Phys. Lett.* **98**, 081106 (2011).

9. Liu, Z., Dong, N., Jiang, P., Wang, K., Wang, J. & Chen, Y. Reduced graphene oxide chemically modified with aggregation-induced emission polymer for solid-state

optical limiter. *Chem. Eur. J.* **24**, 19317-19322（2018）.

10. Shi, M. et al. Donor-acceptor type blends composed of black phosphorus and C-60 for solid-state optical limiters. *Chem. Commun.* **54** , 366-369 (2018).

11. Sun, Y. et al. Superb nonlinear absorption of triphenylene-based metal-organic frameworks associated with abundant metal d electrons. *Adv. Opt. Mater.* **9**, 2100622 (2021).

12. Niu, R.-J. et al. Morphology-dependent third-order optical nonlinearity of a 2D Co-based metal-organic framework with a porphyrinic skeleton. *Chem. Commun.* **55**, 4873-4876 (2019).

13. Xu, B.-W. et al. Similarities and differences between Mn(II) and Zn(II) coordination polymers supported by porphyrin-based ligands: synthesis, structures and nonlinear optical properties. *Dalton. Trans.* **49**, 12622-12631 (2020).

14. Du, Y. et al. Covalent functionalization of graphene oxide with porphyrin and porphyrin incorporated polymers for optical limiting. *Phys. Chem. Chem. Phys.* **19**, 2252-2260 (2017).

15. Zheng, C. et al. Facile control of metal nanoparticles from isolated nanoparticles to aggregated clusters on two-dimensional graphene to form optical limiters. *J. Mater. Chem. C.* **5**, 11579-11589 (2017).

16. Li, J. et al. Tetrazine chromophore-based metal-organic frameworks with unusual configurations: synthetic, structural, theoretical, fluorescent, and nonlinear optical studies. *Chem. Eur. J.* **21**, 7914-7926 (2015).
